# Regulation of Iron Homeostasis and Related Diseases

**DOI:** 10.1155/2020/6062094

**Published:** 2020-05-02

**Authors:** Yikun Li, Xiali Huang, Jingjing Wang, Ruiling Huang, Dan Wan

**Affiliations:** ^1^Key Laboratory of Agro-Ecological Processes in Subtropical Region, Hunan Research Center of Livestock & Poultry Sciences, South-Central Experimental Station of Animal Nutrition and Feed Science in Ministry of Agriculture, Institute of Subtropical Agriculture, The Chinese Academy of Sciences, Changsha, Hunan 410125, China; ^2^University of Chinese Academy of Sciences, Beijing 100049, China; ^3^Department of Animal Nutrition and Feed Science, College of Animal Science and Technology, Huazhong Agricultural University, Wuhan 430070, China

## Abstract

The liver is the organ for iron storage and regulation; it senses circulating iron concentrations in the body through the BMP-SMAD pathway and regulates the iron intake from food and erythrocyte recovery into the bloodstream by secreting hepcidin. Under iron deficiency, hypoxia, and hemorrhage, the liver reduces the expression of hepcidin to ensure the erythropoiesis but increases the excretion of hepcidin during infection and inflammation to reduce the usage of iron by pathogens. Excessive iron causes system iron overload; it accumulates in never system and damages neurocyte leading to neurodegenerative diseases such as Parkinson's syndrome. When some gene mutations affect the perception of iron and iron regulation ability in the liver, then they decrease the expression of hepcidin, causing hereditary diseases such as hereditary hemochromatosis. This review summarizes the source and utilization of iron in the body, the liver regulates systemic iron homeostasis by sensing the circulating iron concentration, and the expression of hepcidin regulated by various signaling pathways, thereby understanding the pathogenesis of iron-related diseases.

## 1. Introduction

Iron is the maximum trace element in the body. As a transition metal, iron readily donates and accepts electrons to participate in biologic processes like oxygen transport, mitochondrial respiration, nucleic acid replication, intermediary, xenobiotic metabolism, and cell signaling [1]. Iron is so important is that its deficiency is one of the major risk factors for disability and death worldwide, and it is estimated to affect 2 billion people [2, 3]. On the other hand, excessive iron is harmful; it damages the liver and the brain, causing oxidative stress on the nerve to cause neurodegenerative diseases such as Parkinson's syndrome. Mutations in multiple iron-regulated pathways lead to heredity iron overload diseases like hereditary hemochromatosis (HH) and iron-refractory iron deficiency anemia (IRIDA) [4].

## 2. Absorption of Iron in the Food and Cellular Iron Acquisition

Dietary iron includes the heme iron and nonheme iron; 90% of them are nonheme iron, mainly present as the form of Fe(OH)_3_ complexation. Nonheme dietary iron is absorption at the brush border of duodenal enterocytes and exhibited diurnal rhythms [5]. The cytochrome b (Dcytb) on the duodenal enterocyte membrane reduced Fe^3+^ to Fe^2+^, then the Fe^2+^ through the divalent metal transporter 1 (DMT1) on the membrane into the cell. The heme iron absorption mainly uptakes by the heme carrier protein 1 (HCP-1) [6, 7]. When the heme gets into the cell, it is degraded into iron, carbon monoxide, and biliverdin by heme oxygenase 1 or 2 (HO-1/2) [8]. Intracellular iron is efflux to the extracellular by the ferroportin1(FPN1), the only iron transmembrane efflux protein in vertebrate cells [9–11]. Excess cellular iron is stored in ferritin, which has a large cavity to store thousands of iron atoms; it prevents dissociative iron from causing oxidative damage to cells [12]. After the Fe^2+^ efflux into the circulation, it oxidized to Fe^3+^ by the ferroxidases such as hephaestin (HEPH) or its homologue ceruloplasmin (CP) [13, 14] and succeedingly loaded onto the transferrin (Tf) and transported by the bloodstream.

The majority of the blood iron participates in hematopoiesis in the bone marrow, and a minor part transports to the liver. The liver is the essential organ for the body to store the iron, and the iron in hepatocytes is mainly stored in ferritin. For the excess iron, it is engulfed by the Kupffer cells of the reticuloendothelial system and deposited in the system as the form of hemosiderin [15].

**Figure 1:**
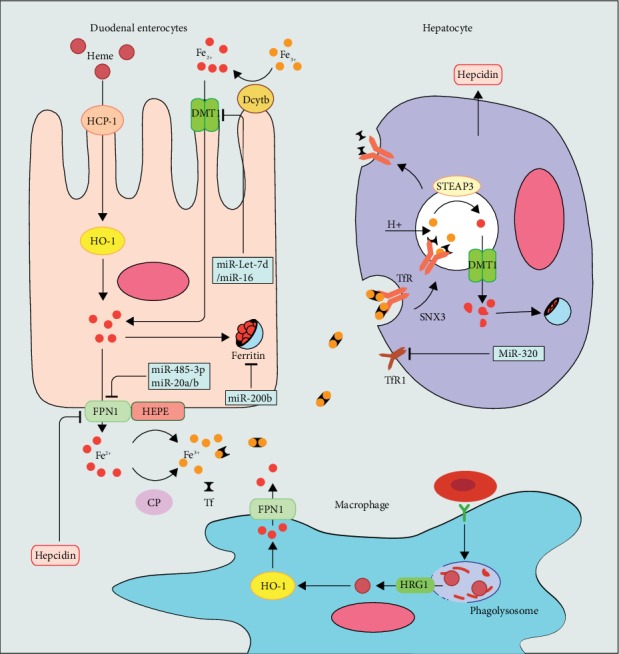
Systemic iron homeostasis.

Iron in the blood binds to the cell surface transferrin receptor (TfR), Tf-Fe/TfR complex sag, and endocytose into the cell, subsequently the conformational of the complex is changed triggered by the acidified endosomes [16, 17], which releases iron from the Tf [18]. The iron in the endosome is restored to Fe^2+^ by prostate six-transmembrane epithelial antigen of prostate 3 (STEAP3) and transported into the cytolymph by DMT1 [19]. The apo-Tf and TfR complex in the endosome are recycled to the cell surface. The sorting nexin 3 (SNX3) is one of the proteins of the phosphoinositide-binding protein family [20], is required for the recycling of Tf/TfR in endocytisis, and increases iron absorption by Tf recycling and bound ability [21]. We summarized the iron absorption and cellular iron acquisition in Figure 1.

### 2.1. Iron Cycle Is Associated with the Production and Clearance of Erythrocyte

In humans, 200 billion red blood cells are producing every day, requiring more than 2 × 10^15^ iron atoms per second to maintain erythropoiesis. The demand for iron is majorly obtained from recycling erythrocytes, so the production and clearance of erythrocytes are critical for iron homeostasis [22].

The erythropoiesis occurs in the erythroblastic island of the late fetal liver and adult bone marrow which surrounds a central macrophage, termed as nurse macrophage. The nurse macrophage promotes erythropoiesis in the erythroblastic island niche [23], phagocytosing the nuclei expelled from erythroid precursor cells in the late stage of erythropoiesis [24]. Other than that, macrophage in the erythroblastic island produces and releases ferritin by exocytosis [25]; then, the ferritin is endocytosed into the erythroblasts [26]. After entering the cell, iron releases from ferritin after acidification and proteolysis, which is used for heme production during the development of erythrocytes [27]. It seems that macrophages provide ferritin to nurture erythroblastic but have others also point out that the transferrin is the sole iron source during erythropoiesis; ferritin endocytosis is just a tiny force for the erythroblastic acquisition iron [28].

While the life of the erythrocyte is about to end or get irreparable damage, the bloodstream takes their last ride to the reticuloendothelial system in the splenic and hepatic. There, it is known that splenic red pulp macrophage cleans up senescent and damaged red blood cells then recycles iron for erythropoiesis after hemoglobin catabolism [29]. There, firstly, the residential macrophage scrutinizes the passaged erythroid [30], then triggered engulf and digest the erythrocytes when macrophages contact to erythrocyte receptors and detect the specific markers on its surface [31], like phosphatidylserine and band 3 [32, 33]. Whereafter, the red blood cell is phagocytosed by macrophages into macrophage phagolysosome, causing hemoglobin breakdown and the heme release [34]. Subsequently, heme in phagolysosome is exported to the cytosol via the heme transporter (HRG1) and is decomposed into iron by HO-1/HO-2 [35, 36], then the iron is utilized by macrophages or effluxed extracellular by FPN1. [8]. Macrophages for the iron cycle are shown in Figure 1.

Fe^3+^ in food is reduced to Fe^2+^ by Dcytb on the duodenal epithelium; it absorbs Fe^2+^ from the intestinal cavity through DMT1. HCP1 intakes heme in food, and HO-1 degrades it into Fe^2+^ in the cytoplasm. Excessive iron storage in the ferritin and other export into the blood through by FPN1; after that, Fe^2+^ is oxidized by CP and HEPH at the basolateral side then loads onto Tf.

Macrophage phagocytosed erythrocytes and releases heme in the phagolysosome. HRG1 exports heme from phagolysosome into the cytosol; then, HO-1 degrades heme into Fe^2+^ and efflux into the bloodstream by FPN1.

Tf-Fe combine with TfR on cytomembrane. SNX3-induce Tf-TfR sag and endocytose into the cell. Acidified endosomes release Fe^3+^ and restored to Fe^2+^ by STEAP3 and Fe^2+^ into the cytoplasm through the DMT1. Apo-Tf and TfR complex is recycled to the cell surface, and Tf is released into the blood.

The miR-Let-7d and miR-16 family decreases DMT1 expression. Hepcidin internalizes and degrades FPN1. miR-485-3p and miR-20b regulate the expression of FPN1. miR-200b induces downregulation of ferritin, and miR-320 suppresses the expression of TfR1.

### 2.2. Hepcidin-FPN1 Axis Sensing and Regulating the Systemic Iron Homeostasis in the Liver

Except for the storage of iron, the liver is the most important organ to regulate the systemic iron homeostasis by secreting the hepcidin. Hepcidin (*HAMP*) is a polypeptide that synthesizes regulatory hormone; it regulates iron homeostasis by combining FPN1 at extracellular to internalize and degrade FPN1 in the lysosome [37]. Iron in the blood loads on the Tf and transports with the bloodstream after being exported from the FPN1. While the concentration of circulating iron floats, hepatocytes sense and regulate hepcidin expression through the BMP/SMAD pathway to regulate the iron output from FPN1 [38]. This way, hepatocyte controls the amount of iron in circulation within the normal range, and unregulated hepcidin in the liver can cause iron deficiency or iron overload.

In the BMP/SMAD pathway, bone morphogenetic protein (BMP) and its coreceptor hemojuvelin (HJV) are the most critical hepcidin that regulate signaling pathway in quantitation [39]. BMP6 is predominantly secreted from liver endothelial cells [40]; its expression is regulated by iron [41], so it reflects the hepatic iron level [42, 43]. BMP6 and HJV together activate the BMP serine threonine kinase receptor- (BMPR-) I/II complex [44, 45]. BMP6/HJV complex as a ligand combines with the BMPR I (Alk2 and Alk3) [46], and BMPR II (ActR2a and BMPR2) [47] promotes phosphorylation downstream BMP media such as SMAD1, SMAD5, and SMAD8 (SMAD1/5/8) [48]. Phosphorylated SMAD1/5/8 combines with the cytoplasmic SMAD4 as an active transcriptional complex and moves into the nucleus; the complex combines with the BMP reaction element (BMP-RE1 and BMP-RE2) and then activates transcription of the *HAMP* [49, 50]. MT-2 (matriptase-2, *TMPRSS6*, transmembrane protease serine 6) is ubiquitously expressed in the liver, invalid of MT-2 due to genetic mutation causes iron-refractory iron deficiency anemia (IRIDA) [51, 52], and MT-2 is also downregulated by iron and BMP6 [53, 54]. HJV is a glycophosphatidylinositol- (GPI-) anchored protein [55]; MT-2 cleavage the membrane HJV (m-HJV) to a form of soluble HJV (s-HJV) to decreases the affinity for BMP6 [56]; thus, the MT-2 expression increases during iron deficiency [57]. However, recent research shows that MT-2 independently cleaves HJV to regulate hepcidin expression, and it also cleaves other components in the BMP/SMAD pathway other than HJV [58]. The furin family of proprotein convertases expressed in the liver also produces s-HJV by cleaving the HJV, but different with MT-2; sHJV generated by furin negatively regulated BMP while the MT-2 only reduces the combination [56]; this process is regulated by iron deficiency or hypoxia [59]. Others like endofin, ATOH8, and SMAD7 also affect the signal transduction of the BMP/SMAD pathway [60–62], which is the perceptron and messenger of iron concentration.

As the iron concentration in the blood, it is sensed by the liver through the Tf-Fe competing with HFE binding to TfR (TfR1/TfR2) on the hepatocyte cytomembrane [63]. The difference in the ability of Tf and HFE to bind to TfR transmits a signal of concentration of the blood iron in hepatocyte [64]. The capability of Tf-Fe combined with TfR1 is stronger than HFE, and Tf-Fe combined with TfR1 is far stronger than TfR2 [65]. While the systemic iron fluxes at a high concentration, the saturation of iron binding to TfR1 and the excessive Tf-Fe binding to TfR2, at the same time, the HFE having no choice but combining with TfR2 or free on the cell surface, both of these states transmit signals to stimulate the expression of hepcidin. While the systemic iron fluxes, the high concentration of the Tf-Fe saturated binding to all TfR1 and the rest excessive Tf-Fe binding to TfR2, at the same time, the HFE only binds to TfR2 or dissociation on the cytomembrane, the combination of TfR2 with either Tf-Fe or HFE can transmit the signals to stimulate the expression of hepcidin [66]. When the iron in circulating decreases, TfR1 combines all of Tf-Fe and partial HFE, uncombined TfR2 weaken the effects of the stimulation and decreasing the expression of hepcidin to augment the intestinal iron absorption [55].

**Figure 2:**
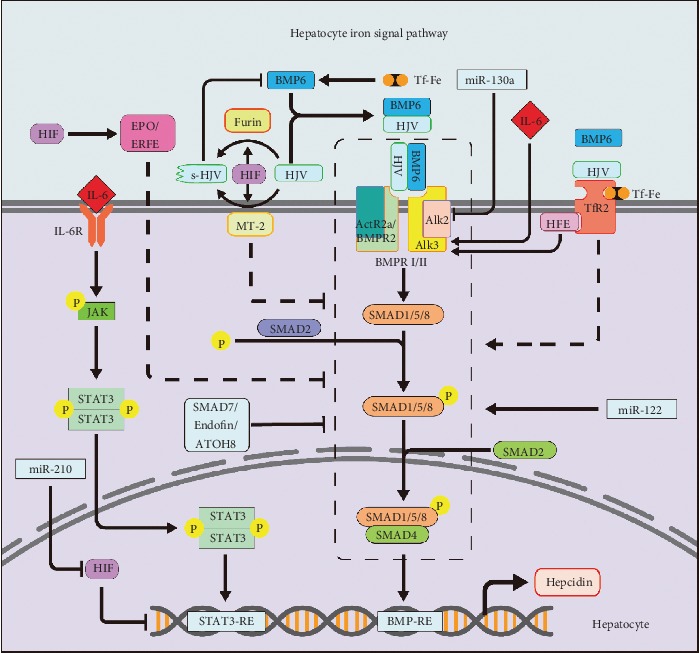
Hepatocyte pathways regulate iron homeostasis.

It is not completely clear how TfR2, HFE, and HJV affect hepcidin expression, but there have been experiments shown in *HFE* and *TfR2* knockout mice that the conduction of the BMP/SMAD signaling pathway was impaired [67, 68]. Recent research shows that the noncompetitive binding of HFE and TfR2 to HJV causes changes in hepcidin expression [55]; in addition, HFE also has the ability to regulate the BMP/SMAD signaling pathway by binding to ALK3 [69]. Neogenin is also involved in the regulation of the hepcidin by being a scaffold of binding HJV and ALK3 [22, 70]; it increases the stability of the HJV protein and suppressing HJV secretion [71]. Besides that, neogenin inhibits the BMP-2-induced phosphorylation of the Smad1/5/8 [72] and facilitates the cleavage of HJV by matriptase-2 or furin [70, 73]. There are others pointout the HJV-neogenin interaction dose not only exist in the liver but also in other tissues [70]. Signal pathways in hepatocytes regulate hepcidin expression as shown in Figure 2.

BMP/SMAD signaling pathway: BMP6 and its coreceptor HJV activate BMPR I/II, leading to phosphorylation of SMAD (1/5/8) and complexes with SMAD4 as an active transcriptional complex. The complex combines with the BMP-RE on *HAMP* then activates transcription of the hepcidin. SMAD2 promotes the phosphorylation of SMAD (1/5/8). SMAD7, endofin, and ATOH8 reduce the signaling of BMP/SMAD. HJV is cleaved by MT-2 and furin to reduced binding capacity to BMP6. miR-130a and miR-122 inhibit AIL2 and BMP/SMAD to regulate the expression of hepcidin.

High concentrations of Tf-Fe induce HFE and Tf-Fe which combine with TfR2 and HJV together to promote BMP/SMAD signaling pathway. HFE interacts with ALK3 increasing hepcidin excrete.

Hypoxia induces the HIF-determined EPO/ERFE concentration in the blood circulation; all of them increase the systemic iron concentration through the BMP/SMAD pathway. HIF promotes MT2 and furin to cleavage HJV, and miR-210 inhibits it to reduce hepcidin expression. Iron increases BMP6 expression.

In inflammation, IL-6 combines with its receptor IL-6R to activate the JAK, triggering the phosphorylates of STAT3 that forms as a complex move into the nucleus and promotes the transcription of *HAMP*. IL6 increases the BMP/SMAD pathway by promoting ALK3.

### 2.3. Role of Inflammation, Hypoxia, and MicroRNA in Iron Regulation

Infection and inflammation induce hepcidin production [74], which inhibits iron efflux from intestinal and promotes iron chelation in macrophages, thus reducing the concentration of blood iron [75]. During inflammation, the secretion of proinflammatory cytokines (such as IL-6) increases. Interleukin-6 (IL-6) is one kind of cytokine that regulates the transcription of hepcidin [76]. It combines with the IL-6 receptor (IL-6R) on the membrane, then activates JAK and phosphorylates STAT3 protein in hepatocytes. The phosphorylated STAT3 protein moves into the nucleus, regulating the expression of *HAMP* by binding to the STAT3-specific site [77]. IL-6 not only affects hepcidin expression through the JAK/STAT3 pathway but also combines one of the BMPR I receptor Alk3 [78]; this indicates that the JAK-STAT3 pathway has cross action with the BMP/SMAD pathway [79]. In acute inflammatory condition, the stimulation of Toll-like receptor reduces FPN1 in macrophages, blocking the iron excretion from macrophages which is recovered from red blood cells and rapidly induced hypoferremia [80]. Then, the heme, required by the proerythroblast to complete its terminal differentiation stage, is exported from macrophages by FLVCR1 [81]. High hepcidin lowers the pathogens available in iron; it is a strategy to starve the pathogens to limit their growth [82]. But as one of the defensin-like peptide hormones, the innate immunity functions of hepcidin have a connection to antimicrobial peptides and inflammation; perhaps, the role of hepcidin in immunity could bypass iron and be directly related to hosting defense (Figure 2).

The body compensates for the oxygen content by intensifying the erythropoiesis when hypoxia, blood loss, or the other causes. In response to the erythropoiesis, erythropoietin (EPO) is secreted by the kidney. According to the severity of hypoxia, EPO has a hundred times of differing in serum [83], it controls iron absorption, erythroid progenitor cell proliferation, maturation, and survival [84, 85]. Erythroferrone (ERFE) is a soluble protein released by EPO-stimulated erythroid precursors; it suppresses the expression of hepcidin [86]. EPO and ERFE suppress the expression of hepcidin by BMP/SMAD pathway target genes [87–89]. But in the IRIDA, due to the MT-2 restriction, the EPO/ERFE-mediated hepcidin downregulation in the BMP/SMAD pathway is obstructed, the blocked signal transmission leads both the EPO and the ERFE, and hepcidin simultaneously maintained elevated levels even in the patients with anemia [90]. The hypoxia-inducible factor (HIF) is a transcription factor of EPO, and the content of the EPO is completely dependent on HIF-2*α* [91]. HIF-2*α* promotes erythropoiesis, including increases in the production of EPO, which enhances iron uptake and utilization [92]. Therefore, hypoxia increases cthe demand for iron and reduces the expression of hepcidin by HIF and EPO [93]. Hepcidin promoter contains several HIF1 and HIF2 sites, regulating the hepcidin by the hypoxia-oxygen-sensing regulatory pathway [94]. Besides that, HIF participates in the BMP/SMAD pathway by affecting the MT-2 and increasing the furin mRNA level [95, 96] (Figure 2).

MicroRNAs are a class of small noncoding RNAs (~22 nt) that bind to the 3′ untranslated region (3′UTR) of the target messenger RNA (mRNA), thereby negatively regulating gene expression, and many miRNAs are involved in posttranscriptional regulation of iron. miR-485-3p and miR-17 seed family member miR-20a and miR-20b, as the concurrent modulator to regulate the expression of FPN1 [97–99]. miR-Let-7d and miR-16 family (miR-15b, miR-16, miR-195, and miR-497) bind the 3′UTR of *DMT1*-IRE mRNA then decrease DMT1 expression levels, causing iron accumulation in the endosomes, or hoarded in ferritin or used for iron-related proteins [100–102]. MiR-320 is another microRNA related to cellular iron uptake, which inhibits TfR1 expression and prevents cell proliferation [103], and miR-200b induces downregulation of ferritin [104]. In the BMP-SMAD signaling, ALK2 as primary endogenous BMP type I receptors is involved in systemic iron regulation; miR-130a targets 3′UTR of *ALK2* to inhibit BMP-SMAD signaling and the expression of hepcidin; it was upregulated in the iron deficiency mice [105].

In the regulation of hypoxia, *HIF-1α* hypoxia response element-binding site was identified in the promoter of miR-210; the miR-210 is specifically induced by HIF-1*α* during hypoxia [106]. Iron-sulfur cluster scaffold protein (ISCU) is an iron homeostasis essential molecule; iron deficiency induces miR-210 expression through HIF-1*α*, and miR-210 directly inhibits ISCU and TfR to maintain the systemic iron homeostasis [107]. miR-122 is a very important microRNA that is selectively expressed in the liver and participates in a variety of regulation, including maintaining iron homeostasis. It controls hepcidin mRNA transcription by inhibiting the expression of *Hfe*, *Hjv*, and *Bmpr1a* in the liver, thereby preventing iron deficiency [108], thus activating *Hamp* mRNA expression. miRNAs related to iron regulation are summarized in Figures 1 and 2.

### 2.4. Diseases Related to Disorders of Iron Metabolism

#### 2.4.1. Iron Overload Causes Cell Oxidative Damaged-Ferroptosis

Ferroptosis is a form of regulated cell death; unlike other forms of regulated cell death, ferroptosis is unnecessary for the caspases [109]. Ferroptosis is characterized by the overwhelming iron-dependent oxidative injury and accumulation of lipid hydroperoxides to lethal levels. The excessive iron produces ROS (reactive oxygen species) by Fenton reaction in cells. In cells, the ROS has multiple sources; iron and its derivatives are essential for the ROS-producing enzymes.

Ferroptosis is related to amino acid metabolism. Glutathione (GSH) protects cells from oxidative stress damage, but the availability of cysteine limits the GSH biosynthesis [110]; therefore, the cysteine is contributed to protecting cells from oxidative stress. Cysteine is produced by the reduction of cystine which is transported into cells by the cystine/glutamate reverse transport system xc^−^, then for the GSH synthesis. Cells not only rely on the system xc^−^ to import cystine but also bypass the system xc^−^ by the transsulfuration pathway to biosynthesize the cysteine from methionine.

The depletion of GSH inactivation of the GSH peroxidase 4 (GPX4) ultimately causes the ferroptosis. Erastin, an oncogenic RAS-selective lethal small molecule [111], induces ferroptosis by inducing GSH depletion and inactivation of the phospholipid peroxidase GPX4 and inhibits import of cystine [112]. So amino acid metabolism is closely linked to ferroptosis [113]; furthermore, studies on the association of ferroptosis and various diseases have provided a new perspective and have become a new research aspect, such as Parkinson's disease, Alzheimer's disease, Huntington's disease, stroke, apoplexy, ischemia-reperfusion injury, cardiopathy, carcinogenesis, periventricular leukomalacia, and brain injury [110].

#### 2.4.2. Parkinson's Disease

Parkinson's disease (PD) is a progressive neurological disorder, primarily from the death of dopaminergic neurons in the substantia nigra [114]. Studies have shown that Parkinson's disease is caused by biochemical abnormalities, including oxidative stress and mitochondrial dysfunction [115, 116], and in recently, some studies have also shown the correlation between PD and ferroptosis [117].

Iron accumulation in neurons induces oxidative stress by Fenton's reaction generating ROS. ROS induces iron release from mitochondrial iron-sulfur cluster protein and other iron storage proteins; it leads the further ROS generation through Fenton's reaction [118], then the ROS damage DNA and mtDNA by epigenetic mechanism and oxidize protein [118–120]. The most significant characteristic of Parkinson's disease is the progressive degeneration in the substantia nigra, but current research is still incomprehensible why neurodegeneration only exists in certain nuclei while the other iron-accumulation tissue remains unaffected and the mechanism of neurotoxicity [121].

Mitophagy, the spontaneous and selectively autophagic elimination of damaged or dysfunctional mitochondria, is regulated by accumulation of iron, Parkin, and PINK1 (PTEN-induced putative kinase protein 1) and mediated by autophagosomes. There are reports that show the loss of iron in neuronal triggering mitophagy in a PINK1/Parkin-independent manner [122, 123], and in contrast, the accumulation of cellular iron obstructed the mitophagy, so that the cells unable to eliminate the damaged mitochondria to maintain normal physiological status.

PINK1 is stably localized on damaged mitochondria with low membrane potential [124], and the Parkin, an E3 ubiquitin ligase, is selectively recruited from cytosol to dysfunctional mitochondria [125] and liberates the activity of the E3 by the PINK1-dependent mitochondrial localization [124], then Parkin ubiquitination outer mitochondrial membrane proteins to trigger mitophagy [126]. So, the PINK1 and Parkin together sense the distress of mitochondria and selectively target them for degradation [127], and the mutations of PINK1 or Parkin fail to clear damaged mitochondria [128, 129], causing neuronal damage [130], leading to Parkinson's disease [131].

#### 2.4.3. Hereditary Iron Disease

Hereditary hemochromatosis (HH) mainly in Western populations causes iron overloaded. HH is caused by multiple genetic defects like *HFE*, *TfR2*, *HJV*, *TMPRSS6*, *FPN1*, and *HAMP*. According to different mutant genes, HH is divided into HFE hemochromatosis (type1), juvenile hemochromatosis (type 2), TfR2 hemochromatosis (type 3), and ferroportin hemochromatosis.

The majority of the HH is typ1 and typ2; it is due to the homozygosity for the C282Y mutation in the *HFE* and *G320V*, etc. in the *HJV* genes [132–135]. *HFE* and *HJV* mutations alone or simultaneously affect the expression of hepcidin through the BMP/SMAD pathway. In type 3 HH, the mutation was identified on human chromosome 7q22 homozygous recessive *Y250X* in *TfR2* [136]; type 3 HH is less severe than typ1 and typ2 HH. Type 3 HH pathogenesis is demonstrated in the mutation experiment of mice; the mutation of TfR2 caused the inability of TF and HFE to bind to it, weakened the signal transmission, and resulted in the downregulation of hepcidin expression [137], which ultimately caused iron overload in multiple organs. As a receptor for hepcidin, *FPN1* has *C326* residue and is necessary for the binding of hepcidin [138]. Ferroportin hemochromatosis is associated with the mutation of C326 residue, it is an autosomal dominant genetic disease with similar clinical and phenotypic features to other HH, and the mutation of *C326* suffices to cause FPN1 resistance of hepcidin [138, 139]. The loss-of-function mutation of *TMPRSS6* causes IRIDA, and its molecular basis was first identified in 2008 [140, 141]. Microcytic hypochromic anemia, low Tf saturation, and excessive hepcidin are the main characteristic of *IRIDA*; however, oral iron supplementation is futile in relieving the symptoms. In the *IRIDA*, the most frequent mutation is *S304L*; besides that, 40 different mutations in the *TMPRSS6* gene have been described including *K225E*, *K253E*, *G228D*, *R446W*, *V736A*, and *V795I* [142], but the latest research shows that *ALK2* gene mutation is also involved in the IRIDA [143]. Including the gene mutations mentioned above, we summarized the various genetic variations that caused hereditary iron disease in Table 1.

#### 2.4.4. Perspectives

As one of the most important elements in the body, after the decades of research, we have been clear about the effect of the liver on iron metabolism and regulation, but we are still constantly discovering new methods to affect iron metabolism directly or indirectly. As the secretory organ of hepcidin, the study of microRNA and gene mutations has opened a new horizon for iron regulation in hepatocyte. The discovery of more potential regulators raises more awareness of iron metabolism, and more drugs can be developed to treat iron-related diseases, such as inhibitors or agonist of key genes. Due to the importance of iron in the body, the molecular mechanism of iron sensing and regulation and its interaction needed to fully comprehend.

## Figures and Tables

**Table 1:** Genetic mutation causes iron metabolism disorders.

Protein	Gene	Mutation site	Downstream effect	Phenotype	Reference
HFE	HFE	C282Y	Iron concentration perception	Regulation hepcidin expression by binding TfR	[133]
TfR2	TFR2	Y250X	Tf, HFE receptor	C282Y homozygote modifier	[144]
HJV	HJV	G320V, etc.	Activate BMP-SMAD	Regulation hepcidin expression	[134]
MT2	TMPRSS6	A736V	Cleavage HJV	Determining protease activity, influences the hepcidin response to iron	[140]
FPN1	SLC40A1	C326S	Cellular iron effluxion	Resistance combine hepcidin	*[* 145, 146*]*

## References

[1] Dev S., Babitt J. L. (2017). Overview of iron metabolism in health and disease. *Hemodialysis International*.

[2] Allen L., Benoist B. D. (2006). *WHO Guidelines on Food Fortification with Micronutrients*.

[3] Wan D., Wu Q., Ni H., Liu G., Ruan Z., Yin Y. (2019). Treatments for iron deficiency (ID): prospective organic iron fortification. *Current Pharmaceutical Design*.

[4] Pietrangelo A. (2017). Ferroportin disease: pathogenesis, diagnosis and treatment. *Haematologica*.

[5] Zhang Y., Wan D., Zhou X. (2017). Diurnal variations in iron concentrations and expression of genes involved in iron absorption and metabolism in pigs. *Biochemical and Biophysical Research Communications*.

[6] Shayeghi M., Latunde-Dada G. O., Oakhill J. S. (2005). Identification of an intestinal heme transporter. *Cell*.

[7] Krishnamurthy P., Xie T., Schuetz J. D. (2007). The role of transporters in cellular heme and porphyrin homeostasis. *Pharmacology & Therapeutics*.

[8] Gottlieb Y., Truman M., Cohen L. A., Leichtmann-Bardoogo Y., Meyron-Holtz E. G. (2012). Endoplasmic reticulum anchored heme-oxygenase 1 faces the cytosol. *Haematologica*.

[9] Troadec M. B., Ward D. M., Lo E., Kaplan J., de Domenico I. (2010). Induction of FPN1 transcription by MTF-1 reveals a role for ferroportin in transition metal efflux. *Blood*.

[10] Donovan A., Lima C. A., Pinkus J. L. (2005). The iron exporter ferroportin/Slc40a1 is essential for iron homeostasis. *Cell Metabolism*.

[11] Donovan A., Brownlie A., Zhou Y. (2000). Positional cloning of zebrafish ferroportin1 identifies a conserved vertebrate iron exporter. *Nature*.

[12] Harrison P. M., Arosio P. (1996). The ferritins: molecular properties, iron storage function and cellular regulation. *Biochimica et Biophysica Acta-Bioenergetics*.

[13] Chen H., Attieh Z. K., Su T. (2004). Hephaestin is a ferroxidase that maintains partial activity in sex-linked anemia mice. *Blood*.

[14] Hellman N. E., Gitlin J. D. (2002). Ceruloplasmin metabolism and function. *Annual Review of Nutrition*.

[15] Hankins J. S., Smeltzer M. P., McCarville M. (2010). Patterns of liver iron accumulation in patients with sickle cell disease and thalassemia with iron overload. *European Journal of Haematology*.

[16] Kawabata H., Germain R. S., Vuong P. T., Nakamaki T., Said J. W., Koeffler H. P. (2000). Transferrin receptor 2-alpha supports cell growth both in iron-chelated cultured cells and in vivo. *Journal of Biological Chemistry*.

[17] Wally J., Halbrooks P. J., Vonrhein C. (2006). The crystal structure of iron-free human serum transferrin provides insight into inter-lobe communication and receptor binding. *Journal of Biological Chemistry*.

[18] Giannetti A. M., Halbrooks P. J., Mason A. B., Vogt T. M., Enns C. A., Björkman P. J. (2005). The molecular mechanism for receptor-stimulated iron release from the plasma iron transport protein transferrin. *Structure*.

[19] Ohgami R. S., Campagna D. R., Greer E. L. (2005). Identification of a ferrireductase required for efficient transferrin- dependent iron uptake in erythroid cells. *Nature Genetics*.

[20] Cullen P. J., Korswagen H. C. (2012). Sorting nexins provide diversity for retromer-dependent trafficking events. *Nature Cell Biology*.

[21] Chen C., Garcia-Santos D., Ishikawa Y. (2013). Snx3 regulates recycling of the transferrin receptor and iron assimilation. *Cell Metabolism*.

[22] Muckenthaler M. U., Rivella S., Hentze M. W., Galy B. (2017). A red carpet for iron metabolism. *Cell*.

[23] Korolnek T., Hamza I. (2015). Macrophages and iron trafficking at the birth and death of red cells. *Blood*.

[24] Bessis M. C., Breton-Gorius J. (1962). Iron metabolism in the bone marrow as seen by electron Microscopy: a critical review. *Blood*.

[25] Leimberg M. J., Prus E., Konijn A. M., Fibach E. (2008). Macrophages function as a ferritin iron source for cultured human erythroid precursors. *Journal of Cellular Biochemistry*.

[26] Leimberg J. M., Prus E., Link G., Fibach E., Konijn A. M. (2008). Iron-chelator complexes as iron sources for early developing human erythroid precursors. *Translational Research*.

[27] Li L., Fang C. J., Ryan J. C. (2010). Binding and uptake of H-ferritin are mediated by human transferrin receptor-1. *Proceedings of the National Academy of Sciences of the United States of America*.

[28] Hentze M. W., Muckenthaler M. U., Galy B., Camaschella C. (2010). Two to tango: regulation of mammalian iron metabolism. *Cell*.

[29] de Back D. Z., Kostova E. B., van Kraaij M., van den Berg T. K., van Bruggen R. (2014). Of macrophages and red blood cells; a complex love story. *Frontiers in Physiology*.

[30] Mebius R. E., Kraal G. (2005). Structure and function of the spleen. *Nature Reviews Immunology*.

[31] Knutson M., Wessling-Resnick M. (2003). Iron metabolism in the reticuloendothelial system. *Critical Reviews in Biochemistry and Molecular Biology*.

[32] Connor J., Pak C. C., Schroit A. J. (1994). Exposure of phosphatidylserine in the outer leaflet of human red blood cells. Relationship to cell density, cell age, and clearance by mononuclear cells. *Journal of Biological Chemistry*.

[33] Low P., Waugh S., Zinke K., Drenckhahn D. (1985). The role of hemoglobin denaturation and band 3 clustering in red blood cell aging. *Science*.

[34] Bratosin D., Mazurier J., Tissier J. P. (1998). Cellular and molecular mechanisms of senescent erythrocyte phagocytosis by macrophages. A review. *Biochimie*.

[35] White C., Yuan X., Schmidt P. J. (2013). HRG1 is essential for heme transport from the phagolysosome of macrophages during erythrophagocytosis. *Cell Metabolism*.

[36] Yanatori I., Tabuchi M., Kawai Y., Yasui Y., Akagi R., Kishi F. (2010). Heme and non-heme iron transporters in non-polarized and polarized cells. *BMC Cell Biology*.

[37] Nemeth E., Tuttle M. S., Powelson J. (2004). Hepcidin regulates cellular iron efflux by binding to ferroportin and inducing its internalization. *Science*.

[38] Hollerer I., Bachmann A., Muckenthaler M. U. (2017). Pathophysiological consequences and benefits of HFE mutations: 20 years of research. *Haematologica*.

[39] Parrow N. L., Fleming R. E., Cousins R. J. (2014). Bone morphogenetic proteins as regulators of iron metabolism. *Annual Review of Nutrition*.

[40] Canali S., Zumbrennen-Bullough K. B., Core A. B. (2017). Endothelial cells produce bone morphogenetic protein 6 required for iron homeostasis in mice. *Blood*.

[41] Ramos E., Kautz L., Rodriguez R. (2011). Evidence for distinct pathways of hepcidin regulation by acute and chronic iron loading in mice. *Hepatology*.

[42] Daher R., Kannengiesser C., Houamel D. (2016). Heterozygous mutations in BMP6 pro-peptide lead to inappropriate hepcidin synthesis and moderate iron overload in humans. *Gastroenterology*.

[43] Latour C., Besson-Fournier C., Meynard D. (2016). Differing impact of the deletion of hemochromatosis-associated molecules HFE and transferrin receptor-2 on the iron phenotype of mice lacking bone morphogenetic protein 6 or hemojuvelin. *Hepatology*.

[44] Steinbicker A. U., Bartnikas T. B., Lohmeyer L. K. (2011). Perturbation of hepcidin expression by BMP type I receptor deletion induces iron overload in mice. *Blood*.

[45] Babitt J. L., Huang F. W., Wrighting D. M. (2006). Bone morphogenetic protein signaling by hemojuvelin regulates hepcidin expression. *Nature Genetics*.

[46] Yu P. B., Hong C. C., Sachidanandan C. (2008). Dorsomorphin inhibits BMP signals required for embryogenesis and iron metabolism. *Nature Chemical Biology*.

[47] Mayeur C., Leyton P. A., Kolodziej S. A., Yu B., Bloch K. D. (2014). BMP type II receptors have redundant roles in the regulation of hepatic hepcidin gene expression and iron metabolism. *Blood*.

[48] Canali S., Vecchi C., Garuti C., Montosi G., Babitt J. L., Pietrangelo A. (2016). The SMAD pathway is required for hepcidin response during endoplasmic reticulum stress. *Endocrinology*.

[49] Casanovas G., Mleczko-Sanecka K., Altamura S., Hentze M. W., Muckenthaler M. U. (2009). Bone morphogenetic protein (BMP)-responsive elements located in the proximal and distal hepcidin promoter are critical for its response to HJV/BMP/SMAD. *Journal of Molecular Medicine*.

[50] Mayeur C., Kolodziej S. A., Wang A. (2015). Oral administration of a bone morphogenetic protein type I receptor inhibitor prevents the development of anemia of inflammation. *Haematologica*.

[51] Silvestri L., Pagani A., Nai A., de Domenico I., Kaplan J., Camaschella C. (2008). The serine protease matriptase-2 (TMPRSS6) inhibits hepcidin activation by cleaving membrane hemojuvelin. *Cell Metabolism*.

[52] Béliveau F., Tarkar A., Dion S. P. (2019). Discovery and development of TMPRSS6 inhibitors modulating hepcidin levels in human hepatocytes. *Cell Chemical Biology*.

[53] Meynard D., Vaja V., Sun C. C. (2011). Regulation of TMPRSS6 by BMP6 and iron in human cells and mice. *Blood*.

[54] Zhao N., Nizzi C. P., Anderson S. A. (2015). Low intracellular iron increases the stability of matriptase-2. *Journal of Biological Chemistry*.

[55] D'Alessio F., Hentze M. W., Muckenthaler M. U. (2012). The hemochromatosis proteins HFE, TfR2, and HJV form a membrane-associated protein complex for hepcidin regulation. *Journal of Hepatology*.

[56] Maxson J. E., Chen J., Enns C. A., Zhang A. S. (2010). Matriptase-2- and proprotein convertase-cleaved forms of hemojuvelin have different roles in the down-regulation of hepcidin expression. *Journal of Biological Chemistry*.

[57] Zhang A. S., Anderson S. A., Meyers K. R., Hernandez C., Eisenstein R. S., Enns C. A. (2007). Evidence that inhibition of hemojuvelin shedding in response to iron is mediated through neogenin. *Journal of Biological Chemistry*.

[58] Wahedi M., Wortham A. M., Kleven M. D. (2017). Matriptase-2 suppresses hepcidin expression by cleaving multiple components of the hepcidin induction pathway. *Journal of Biological Chemistry*.

[59] Silvestri L., Pagani A., Camaschella C. (2008). Furin-mediated release of soluble hemojuvelin: a new link between hypoxia and iron homeostasis. *Blood*.

[60] Goh J. B., Wallace D. F., Hong W., Subramaniam V. N. (2015). Endofin, a novel BMP-SMAD regulator of the iron-regulatory hormone, hepcidin. *Scientific Reports*.

[61] Upanan S., McKie A. T., Latunde-Dada G. O. (2017). Hepcidin suppression in *β*-thalassemia is associated with the down-regulation of atonal homolog 8. *International Journal of Hematology*.

[62] Lai D., Teng F., Hammad S. (2018). Hepatic Smad7 overexpression causes severe iron overload in mice. *Blood*.

[63] Kawabata H. (2019). Transferrin and transferrin receptors update. *Free Radical Biology and Medicine*.

[64] Kleven M. D., Jue S., Enns C. A. (2018). Transferrin receptors TfR1 and TfR2 bind transferrin through differing mechanisms. *Biochemistry*.

[65] Schmidt P. J., Toran P. T., Giannetti A. M., Bjorkman P. J., Andrews N. C. (2008). The transferrin receptor modulates Hfe-dependent regulation of hepcidin expression. *Cell Metabolism*.

[66] Gao J., Chen J., Kramer M., Tsukamoto H., Zhang A.-S., Enns C. A. (2009). Interaction of the hereditary hemochromatosis protein HFE with transferrin receptor 2 is required for transferrin-induced hepcidin expression. *Cell Metabolism*.

[67] Wallace D. F., Summerville L., Crampton E. M., Frazer D. M., Anderson G. J., Subramaniam V. N. (2009). Combined deletion of Hfe and transferrin receptor 2 in mice leads to marked dysregulation of hepcidin and iron overload. *Hepatology*.

[68] Kent P., Wilkinson N., Constante M. (2015). Hfe and Hjv exhibit overlapping functions for iron signaling to hepcidin. *Journal of Molecular Medicine*.

[69] Traeger L., Enns C. A., Krijt J., Steinbicker A. U. (2018). The hemochromatosis protein HFE signals predominantly via the BMP type I receptor ALK3 in vivo. *Communications Biology*.

[70] Zhao N., Maxson J. E., Zhang R. H., Wahedi M., Enns C. A., Zhang A.-S. (2016). Neogenin facilitates the induction of hepcidin expression by hemojuvelin in the liver. *Journal of Biological Chemistry*.

[71] Lee D. H., Zhou L. J., Zhou Z. (2010). Neogenin inhibits HJV secretion and regulates BMP-induced hepcidin expression and iron homeostasis. *Blood*.

[72] Hagihara M., Endo M., Hata K. (2011). Neogenin, a receptor for bone morphogenetic proteins. *Journal of Biological Chemistry*.

[73] Enns C. A., Ahmed R., Zhang A. S. (2012). Neogenin interacts with matriptase-2 to facilitate hemojuvelin cleavage. *Journal of Biological Chemistry*.

[74] Wang C. Y., Babitt J. L. (2016). Hepcidin regulation in the anemia of inflammation. *Current Opinion in Hematology*.

[75] Ganz T., Nemeth E. (2016). Iron balance and the role of hepcidin in chronic kidney disease. *Seminars in Nephrology*.

[76] Nemeth E., Rivera S., Gabayan V. (2004). IL-6 mediates hypoferremia of inflammation by inducing the synthesis of the iron regulatory hormone hepcidin. *The Journal of Clinical Investigation*.

[77] Wrighting D. M., Andrews N. C. (2006). Interleukin-6 induces hepcidin expression through STAT3. *Blood*.

[78] Mayeur C., Lohmeyer L. K., Leyton P. (2014). The type I BMP receptor Alk3 is required for the induction of hepatic hepcidin gene expression by interleukin-6. *Blood*.

[79] Falzacappa M. V. V., Casanovas G., Hentze M. W., Muckenthaler M. U. (2008). A bone morphogenetic protein (BMP)-responsive element in the hepcidin promoter controls HFE2-mediated hepatic hepcidin expression and its response to IL-6 in cultured cells. *Journal of Molecular Medicine*.

[80] Guida C., Altamura S., Klein F. A. (2015). A novel inflammatory pathway mediating rapid hepcidin-independent hypoferremia. *Blood*.

[81] Keel S. B., Doty R. T., Yang Z. (2008). A heme export protein is required for red blood cell differentiation and iron homeostasis. *Science*.

[82] Vyoral D., Petrak J. (2017). Therapeutic potential of hepcidin − the master regulator of iron metabolism. *Pharmacological Research*.

[83] Ebert B. L., Bunn H. F. (1999). Regulation of the erythropoietin gene. *Blood*.

[84] Artuso I., Pettinato M., Nai A. (2019). Transient decrease of serum iron after acute erythropoietin treatment contributes to hepcidin inhibition by ERFE in mice. *Haematologica*.

[85] Rossert J., Eckardt K. U. (2005). Erythropoietin receptors: their role beyond erythropoiesis. *Nephrology Dialysis Transplantation*.

[86] Kautz L., Jung G., Valore E. V., Rivella S., Nemeth E., Ganz T. (2014). Identification of erythroferrone as an erythroid regulator of iron metabolism. *Nature Genetics*.

[87] Aschemeyer S., Gabayan V., Ganz T., Nemeth E., Kautz L. (2017). Erythroferrone and matriptase-2 independently regulate hepcidin expression. *American Journal of Hematology*.

[88] Arezes J., Foy N., McHugh K. (2018). Erythroferrone inhibits the induction of hepcidin by BMP6. *Blood*.

[89] Wang C. Y., Core A. B., Canali S. (2017). Smad1/5 is required for erythropoietin-mediated suppression of hepcidin in mice. *Blood*.

[90] Nai A., Rubio A., Campanella A. (2016). Limiting hepatic Bmp-Smad signaling by matriptase-2 is required for erythropoietin-mediated hepcidin suppression in mice. *Blood*.

[91] Kapitsinou P. P., Liu Q., Unger T. L. (2010). Hepatic HIF-2 regulates erythropoietic responses to hypoxia in renal anemia. *Blood*.

[92] Haase V. H. (2010). Hypoxic regulation of erythropoiesis and iron metabolism. *American Journal of Physiology-Renal Physiology*.

[93] Nicolas G., Chauvet C., Viatte L. (2002). The gene encoding the iron regulatory peptide hepcidin is regulated by anemia, hypoxia, and inflammation. *Journal of Clinical Investigation*.

[94] Safran M., Kaelin W. G. (2003). HIF hydroxylation and the mammalian oxygen-sensing pathway. *Journal of Clinical Investigation*.

[95] McMahon S., Grondin F., McDonald P. P., Richard D. E., Dubois C. M. (2005). Hypoxia-enhanced expression of the proprotein convertase furin is mediated by hypoxia-inducible Factor-1. *Journal of Biological Chemistry*.

[96] Lakhal S., Schödel J., Townsend A. R. M., Pugh C. W., Ratcliffe P. J., Mole D. R. (2011). Regulation of type II transmembrane serine proteinase TMPRSS6 by hypoxia-inducible factors: new link between hypoxia signaling and iron homeostasis. *Journal of Biological Chemistry*.

[97] Sangokoya C., Doss J. F., Chi J. T. (2013). Iron-responsive miR-485-3p regulates cellular iron homeostasis by targeting ferroportin. *PLoS Genetics*.

[98] Babu K. R., Muckenthaler M. U. (2016). miR-20a regulates expression of the iron exporter ferroportin in lung cancer. *Journal of Molecular Medicine*.

[99] Jiang S., Fang X., Liu M., Ni Y., Ma W., Zhao R. (2019). MiR-20b down-regulates intestinal ferroportin expression in vitro and in vivo. *Cell*.

[100] Andolfo I., de Falco L., Asci R. (2010). Regulation of divalent metal transporter 1 (DMT1) non-IRE isoform by the microRNA let-7d in erythroid cells. *Haematologica*.

[101] Hou W., Tian Q., Steuerwald N. M., Schrum L. W., Bonkovsky H. L. (2012). The let-7 microRNA enhances heme oxygenase-1 by suppressing Bach1 and attenuates oxidant injury in human hepatocytes. *Biochimica Et Biophysica Acta-Gene Regulatory Mechanisms*.

[102] Jiang S., Guo S., Li H., Ni Y., Ma W., Zhao R. (2019). Identification and functional verification of microRNA-16 family targeting intestinal divalent metal transporter 1 (DMT1) in vitro and in vivo. *Frontiers in Physiology*.

[103] Schaar D. G., Medina D. J., Moore D. F., Strair R. K., Ting Y. (2009). miR-320 targets transferrin receptor 1 (CD71) and inhibits cell proliferation. *Experimental Hematology*.

[104] Shpyleva S. I., Tryndyak V. P., Kovalchuk O. (2011). Role of ferritin alterations in human breast cancer cells. *Breast Cancer Research and Treatment*.

[105] Zumbrennen-Bullough K. B., Wu Q., Core A. B. (2014). MicroRNA-130a is up-regulated in mouse liver by iron deficiency and targets the bone morphogenetic protein (BMP) receptor ALK2 to attenuate BMP signaling and hepcidin transcription. *Journal of Biological Chemistry*.

[106] Kulshreshtha R., Ferracin M., Wojcik S. E. (2007). A microRNA signature of hypoxia. *Molecular and Cellular Biology*.

[107] Yoshioka Y., Kosaka N., Ochiya T., Kato T. (2012). Micromanaging iron homeostasis hypoxia-inducible micro-RNA-210 suppresses iron homeostasis-related proteins. *Journal of Biological Chemistry*.

[108] Castoldi M., Vujic Spasic M., Altamura S. (2011). The liver-specific microRNA miR-122 controls systemic iron homeostasis in mice. *Journal of Clinical Investigation*.

[109] Reed J. C., Pellecchia M. (2012). Ironing out cell death mechanisms. *Cell*.

[110] Stockwell B. R., Friedmann Angeli J. P., Bayir H. (2017). Ferroptosis: a regulated cell death nexus linking metabolism, redox biology, and disease. *Cell*.

[111] Dixon S. J., Lemberg K. M., Lamprecht M. R. (2012). Ferroptosis: an iron-dependent form of nonapoptotic cell death. *Cell*.

[112] Yang W. S., SriRamaratnam R., Welsch M. E. (2014). Regulation of ferroptotic cancer cell death by GPX4. *Cell*.

[113] Angeli J. P. F., Shah R., Pratt D. A., Conrad M. (2017). Ferroptosis inhibition: mechanisms and opportunities. *Trends in Pharmacological Sciences*.

[114] Dauer W., Przedborski S. (2003). Parkinson's disease: mechanisms and models. *Neuron*.

[115] Schapira A. H. V. (2008). Mitochondria in the aetiology and pathogenesis of Parkinson's disease. *Lancet Neurology*.

[116] Greenamyre J. T., Hastings T. G. (2004). BIOMEDICINE: Parkinson's--Divergent causes, convergent mechanisms. *Science*.

[117] Do Van B., Gouel F., Jonneaux A. (2016). Ferroptosis, a newly characterized form of cell death in Parkinson's disease that is regulated by PKC. *Neurobiology of Disease*.

[118] Ward R., Zucca F. A., Duyn J. H., Crichton R. R., Zecca L. (2014). The role of iron in brain ageing and neurodegenerative disorders. *The Lancet Neurology*.

[119] Melis J. P. M., van Steeg H., Luijten M. (2013). Oxidative DNA damage and nucleotide excision repair. *Antioxidants & Redox Signaling*.

[120] Kwok J. B. J. (2010). Role of epigenetics in Alzheimer's and Parkinson's disease. *Epigenomics*.

[121] Hare D. J., Double K. L. (2016). Iron and dopamine: a toxic couple. *Brain*.

[122] Allen G. F. G., Toth R., James J., Ganley I. G. (2013). Loss of iron triggers PINK1/Parkin-independent mitophagy. *EMBO Reports*.

[123] Ivatt R. M., Whitworth A. J. (2014). The many faces of mitophagy. *EMBO Reports*.

[124] Matsuda N., Sato S., Shiba K. (2010). PINK1 stabilized by mitochondrial depolarization recruits Parkin to damaged mitochondria and activates latent Parkin for mitophagy. *Journal of Cell Biology*.

[125] Narendra D., Tanaka A., Suen D. F., Youle R. J. (2008). Parkin is recruited selectively to impaired mitochondria and promotes their autophagy. *Journal of Cell Biology*.

[126] Pickrell A. M., Youle R. J. (2015). The roles of PINK1, Parkin, and mitochondrial fidelity in Parkinson's disease. *Neuron*.

[127] Narendra D. P., Jin S. M., Tanaka A. (2010). PINK1 is selectively stabilized on impaired mitochondria to activate Parkin. *PLoS Biology*.

[128] Suen D. F., Narendra D. P., Tanaka A., Manfredi G., Youle R. J. (2010). Parkin overexpression selects against a deleterious mtDNA mutation in heteroplasmic cybrid cells. *Proceedings of the National Academy of Sciences of the United States of America*.

[129] Vives-Bauza C., Zhou C., Huang Y. (2010). PINK1-dependent recruitment of Parkin to mitochondria in mitophagy. *Proceedings of the National Academy of Sciences of the United States of America*.

[130] Valente E. M., Abou-Sleiman P. M., Caputo V. (2004). Hereditary early-onset Parkinson's disease caused by mutations in PINK1. *Science*.

[131] Youle R. J., Narendra D. P. (2011). Mechanisms of mitophagy. *Nature Reviews Molecular Cell Biology*.

[132] Valenti L., Fracanzani A. L., Rametta R. (2012). Effect of the A736V TMPRSS6 polymorphism on the penetrance and clinical expression of hereditary hemochromatosis. *Journal of Hepatology*.

[133] De Falco L., Tortora R., Imperatore N. (2018). The role of TMPRSS6 and HFE variants in iron deficiency anemia in celiac disease. *American Journal of Hematology*.

[134] Kong X., Xie L., Zhu H. (2019). Genotypic and phenotypic spectra of hemojuvelin mutations in primary hemochromatosis patients: a systematic review. *Orphanet Journal of Rare Diseases*.

[135] Pietrangelo A., Caleffi A., Henrion J. (2005). Juvenile hemochromatosis associated with pathogenic mutations of adult hemochromatosis genes. *Gastroenterology*.

[136] Camaschella C., Roetto A., Calì A. (2000). The gene *TFR2* is mutated in a new type of haemochromatosis mapping to 7q22. *Nature Genetics*.

[137] Kawabata H., Fleming R. E., Gui D. (2005). Expression of hepcidin is down-regulated in TfR2 mutant mice manifesting a phenotype of hereditary hemochromatosis. *Blood*.

[138] Fernandes A., Preza G. C., Phung Y. (2009). The molecular basis of hepcidin-resistant hereditary hemochromatosis. *Blood*.

[139] Sham R. L., Phatak P. D., West C., Lee P., Andrews C., Beutler E. (2005). Autosomal dominant hereditary hemochromatosis associated with a novel ferroportin mutation and unique clinical features. *Blood Cells, Molecules & Diseases*.

[140] Nai A., Pagani A., Silvestri L. (2011). TMPRSS6 rs855791 modulates hepcidin transcription in vitro and serum hepcidin levels in normal individuals. *Blood*.

[141] Finberg K. E., Heeney M. M., Campagna D. R. (2008). Mutations in TMPRSS6 cause iron-refractory iron deficiency anemia (IRIDA). *Nature Genetics*.

[142] Beutler E., van Geet C., te Loo D. M. W. M. (2010). Polymorphisms and mutations of human TMPRSS6 in iron deficiency anemia. *Blood Cells Molecules and Diseases*.

[143] Pagani A., Colucci S., Bocciardi R. (2017). A new form of IRIDA due to combined heterozygous mutations of TMPRSS6 and ACVR1A encoding the BMP receptor ALK2. *Blood*.

[144] Roetto A., Totaro A., Piperno A. (2001). New mutations inactivating transferrin receptor 2 in hemochromatosis type 3. *Blood*.

[145] Altamura S., Kessler R., Gröne H. J. (2014). Resistance of ferroportin to hepcidin binding causes exocrine pancreatic failure and fatal iron overload. *Cell Metabolism*.

[146] Theurl M., Song D., Clark E. (2016). Mice with hepcidin-resistant ferroportin accumulate iron in the retina. *FASEB Journal*.

